# Phase–Amplitude Coupling between Theta Rhythm and High-Frequency Oscillations in the Hippocampus of Pigeons during Navigation

**DOI:** 10.3390/ani14030439

**Published:** 2024-01-29

**Authors:** Long Yang, Xi Chen, Lifang Yang, Mengmeng Li, Zhigang Shang

**Affiliations:** 1 School of Electrical and Information Engineering, Zhengzhou University, Zhengzhou 450001, China; longyang_zzu@163.com (L.Y.); cx99325@gmail.com (X.C.); flyer1014@163.com (L.Y.); 2 Henan Key Laboratory of Brain Science and Brain-Computer Interface Technology, Zhengzhou 450001, China; 3 Institute of Medical Engineering Technology and Data Mining, Zhengzhou University, Zhengzhou 450001, China

**Keywords:** cross-frequency interactions, theta, local field potentials, flight, goal directed

## Abstract

**Simple Summary:**

The hippocampus (Hp) is involved in various aspects of spatial cognition during navigation, reflected in multiple neural oscillations across different frequencies. Given the structure homology between the avian and mammalian Hp, does it exhibit similar cross-frequency interaction mechanisms? Unlike rodents, birds can navigate by walking and flying. So, does the avian Hp share the same information interaction under different locomotor modes? In this study, we employed rock pigeons with exceptional spatial navigation abilities and conducted two goal-directed navigational experiments: a ground maze task with a circuitous paradigm and an outdoor homing flight task. These experiments were designed to explore the interactions between different frequency bands in the avian Hp during walking and flying navigation. The study revealed that phase–amplitude coupling (PAC) existed in the avian Hp during the navigational active phases. Meanwhile, the sub-frequency bands modulated by theta oscillations are different in different modes of locomotion. These findings support evidence for cross-frequency interactions in the avian Hp during spatial navigation and contribute to explaining the role of coupling, at least partially, in the ongoing cognitive demands during the navigation process.

**Abstract:**

Navigation is a complex task in which the hippocampus (Hp), which plays an important role, may be involved in interactions between different frequency bands. However, little is known whether this cross-frequency interaction exists in the Hp of birds during navigation. Therefore, we examined the electrophysiological characteristics of hippocampal cross-frequency interactions of domestic pigeons (*Columba livia domestica*) during navigation. Two goal-directed navigation tasks with different locomotor modes were designed, and the local field potentials (LFPs) were recorded for analysis. We found that the amplitudes of high-frequency oscillations in Hp were dynamically modulated by the phase of co-occurring theta-band oscillations both during ground-based maze and outdoor flight navigation. The high-frequency amplitude sub-frequency bands modulated by the hippocampal theta phase were different at different tasks, and this process was independent of the navigation path and goal. These results suggest that phase–amplitude coupling (PAC) in the avian Hp may be more associated with the ongoing cognitive demands of navigational processes. Our findings contribute to the understanding of potential mechanisms of hippocampal PAC on multi-frequency informational interactions in avian navigation and provide valuable insights into cross-species evolution.

## 1. Introduction

Goal-directed navigation is a complex task that involves a range of sensory cues, the storage and retrieval of information, as well as the formulation of plans [1]. The hippocampus (Hp) plays a pivotal role in spatial navigation and memory, with damage to these regions resulting in profound deficits [2,3,4]. Hippocampal theta oscillation is a crucial component of memory formation and the representation of navigational cues in animals [5,6]. This low-frequency rhythm is typically modulated by external sensory inputs and internal cognitive processes [7]. Beyond that, the high-frequency activity in the Hp reflects the synchronicity of neuronal assemblies and the cortical processing of localized areas [8]. A prominent theory is that these oscillations belonging to multiple bands achieve interactions and information transfer through cross-frequency coupling (CFC) [9,10], which may serve as a universal mechanism for executing network-level dynamic computations [11] and reflect higher-order representations of brain states [11,12,13].

Research has indicated that multiple high-frequency amplitudes within the Hp are dynamically modulated by the phase of theta oscillation when rats perform active navigation and decision-making tasks [14]. The amplitude modulation of gamma oscillations enabled by the theta phase [15], commonly known as phase–amplitude coupling (PAC), represents the most extensively studied cross-frequency coupling observed in both rodents and humans. PAC is associated with various physiological and pathological states [16]. In spatial memory tasks performed by humans, increased coupling between the theta (2–5 Hz) phase and gamma amplitude is associated with improved task performance [17]. Furthermore, the amplitude modulated by the theta phase also occurs at a higher frequency band beyond the gamma [11,18], called high-frequency oscillations (HFOs, >100 Hz) [19]. The synchronous activity of the hippocampus in the slow gamma (25–50 Hz) and ripple-band oscillations (100–250 Hz) also plays a crucial role in navigation and memory formation [20,21]. In mammalian Hp, the strongest coupling occurs in the theta (8–12 Hz) phase to HFOs (120–180 Hz) during decision-making and behavioral choices and tends to disappear during the approach-to-goal phase [11]. This suggests that these couplings are at least partially associated with sustained cognitive demands.

Currently, the phenomenon of high-frequency amplitudes modulated by theta phases of Hp in mammals such as rats [14], macaques [22], and humans [17] may be relevant to decision-making, navigation, and long-term working memory. However, there is little knowledge as to whether such cross-frequency interactions exist within the hippocampal structure of flying avians during navigation. Addressing this question could help to unravel the different homology of hippocampal function during navigation between birds and mammals, and shed light on whether cross-frequency interaction in Hp is evolutionarily conservative. The hippocampal structures in birds share similar functional roles and comparable circuit mechanisms with mammals [23,24]. The theta rhythm observed in the avian brain is an ancestral characteristic of hippocampal function [25]. During animal navigation, the activity of place cells in Hp during theta oscillations appears to be well-suited for encoding information about the animal’s current location and local trajectory [26]. Hippocampal theta may also play a role in spatial learning and memory [27], making it a highly significant factor in the formation of spatial memory representations. On the other hand, the activation of high-frequency band activity appears to be associated with higher-level information processing in the navigation process of birds [28]. In our previous studies, we found that domestic pigeons (hereafter, pigeons; *Columba livia domestica*) exhibit the highest inter-channel connectivity strength within the Hp region in the gamma frequency band when performing navigation path adjustments [29]. The above findings support the role of Hp oscillations in local network computations during avian navigation.

Here, we attempted to explore the electrophysiological interaction characteristics between different frequency bands within the pigeon’s Hp under the two navigation modes of walking and flying. To achieve this, we designed two goal-directed spatial navigation tasks for pigeons: a ground maze task with a detour paradigm and an outdoor homing flight task. We simultaneously recorded neural and behavioral data during these tasks. In the tasks, pigeons were trained to navigate to a goal to get a food reward. We intended to investigate whether there exists a cross-frequency interaction within the hippocampal region of pigeons during the navigation. We hypothesized that a pigeon’s Hp was functionally similar to the mammalian Hp and would exhibit PAC during the navigation phase, disappearing as they approached the goal. Furthermore, we designed two navigational modes including flying and walking and tried to test if their cross-frequency interactions would display differences.

## 2. Materials and Methods

### 2.1. Experimental Animals and Surgery

In this study, eight adult pigeons (400–450 g, male and female, and 15 months old) raised in the attic of the School of Electrical Engineering and Information were used. All experimental procedures were conducted following the Animals Act, 2006 (China). The use and care of all animals were approved by the Life Science Ethics Committee of Zhengzhou University.

**Figure 1:**
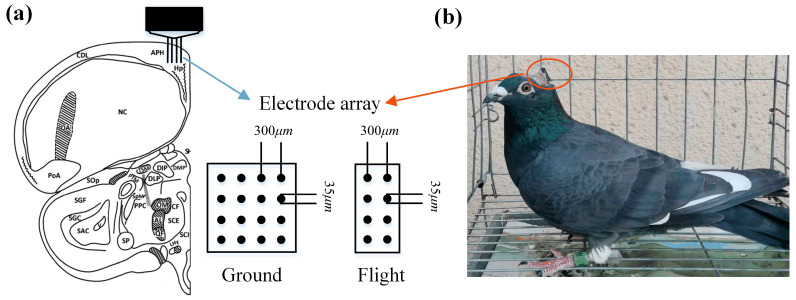
Pigeon Hp microelectrode array implantation. (**a**) Schematic diagram of implanting site and electrode arrays. (**b**) A pigeon after electrode implantation.

The pigeons were familiarized with the maze environment and outdoor release sites separately, two weeks before the surgery. All surgeries were performed after the pigeons were anesthetized via intramuscular injection using 1.5% pentobarbital sodium (0.25 mL/100 g). After ensuring the pigeons were completely anesthetized, they were placed on a stereotaxic apparatus for the procedures. The feathers on the top of the head were carefully trimmed, and then the exposed skin was disinfected using an iodine solution. Next, local anesthesia was administered by injecting lidocaine. The skin in the area was incised, and hydrogen peroxide was used to remove the adhering fascia from the skull. Bleeding was controlled via a hemostatic sponge and a cautery pen. A recording microelectrode array (4×4=16 channels or 2×4=8 channels; platinum iridium alloy; diameter, 35 μm; tip spacing, 300 μm) was implanted into the left Hp (anteroposterior: 4.85–5.10 mm; mediolateral: 1–1.5 mm; dorsoventral: 1.2–1.5 mm). The localization of the Hp was carried out based on the Karten and Hodos stereotaxic coordinates of the pigeon brain [30]. After the surgery, the pigeons were allowed to recover for one week. During this period, the pigeons had access to an ample supply of food, water, and grit. For pigeons participating in the ground maze navigation task, 16-channel electrode arrays were implanted, while for the pigeons participating in the flight navigation task, 8-channel electrode arrays were implanted (Figure 1). The two types of electrodes were identical, with the only difference being the number of channels.

### 2.2. Behavioral Task

For this study, we conducted two goal-directed navigation tasks. We divided the participants into two groups, with each group consisting of four pigeons, performing different tasks. Task 1 involved ground-based maze navigation, and Task 2 involved outdoor flight navigation.

**Figure 2:**
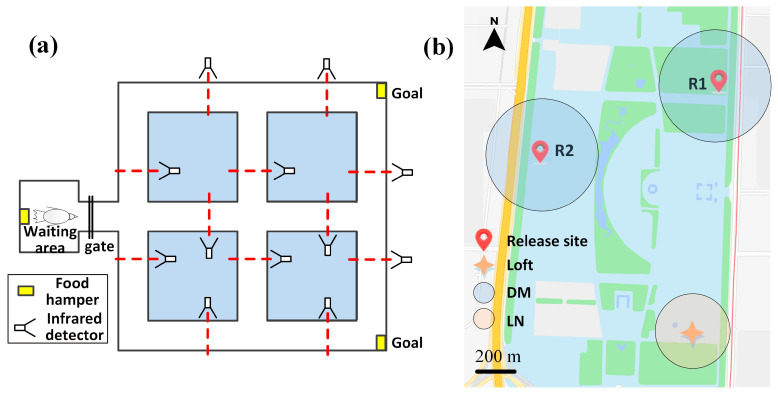
Goal-directed navigational experiment apparatus and environment. (**a**) Diagram of the ground-based maze apparatus. Red markers indicate infrared photobeam positions. (**b**) The release sites for outdoor homing flight navigation. DM: initial decision-making phase; LN: Local navigation around the home.

Task 1: The pigeons were trained to reach specific goal locations within a custom-made maze (Figure 2a). The details were described in our previous description [31]. At both the starting location and the goal locations of the maze, there were food hampers providing food rewards. Once the pigeons could reliably reach the goal location through a stable path, certain sections of the path would be blocked, encouraging the pigeons to form new navigation routes. Along the path, there were infrared detectors distributed to capture the pigeons’ time at different locations within the maze and to intercept signals. By blocking specific paths and changing the goal locations, we successfully guided the pigeons to form three distinct navigation paths.

Task 2: Two release sites (R1 and R2) located approximately 1.5 km away from the pigeon loft in different directions were chosen, forming two flight paths (Figure 2b). Pigeons were trained to fly to the loft from these release sites. For each trajectory, a threshold of 200 m was applied to calculate the distance from the previous trajectory, determining it as the same path. This is because locally experienced birds (having undergone flight training at the same location at least four times) tend to form specific routes within a range of 100–200 m from the previous trajectory [32]. The weather on the release day was clear, with wind speeds below 3 levels. Each pigeon was allowed to be released a maximum of two times per day, with intervals of at least 6 h between each release. The releases took place alternately between the two designated release sites. If the pigeon landed before returning to the loft, the data for that trial were excluded. Before the surgery, the pigeons were trained for five releases at each of the two release sites to ensure they could find their way home.

### 2.3. Data Acquisition

For Task 1, we analyzed 44 sessions recorded from four adult pigeons (P080: 11 sessions; P081: 11 sessions; P082: 12 sessions; P098: 9 sessions) performing a goal-directed spatial task with a detour paradigm. Neural activity was recorded using a 128-channel Cerebus^TM^ Multichannel Acquisition Processor (Blackrock Microsystems, Salt Lake City, UT, USA) at a sampling rate of 2 kHz. The data were amplified with a gain of 300×. Local field potentials (LFPs) were acquired using a 0–250 Hz Butterworth low-pass filter. Data analysis was conducted using custom-written MATLAB scripts (version R2020a). Unless otherwise specified, the analysis was performed on a window of 1000 ms, starting from 250 ms before the pigeons’ initial contact with the infrared detector and extending to 750 ms afterward. The pigeons’ average speed was determined by dividing the distance between infrared detectors by the time taken to pass through specific positions.

For Task 2, we analyzed 17 sessions recorded from four adult pigeons (P010: 4 sessions; P014: 4 sessions; P015: 3 sessions; P017: 6 sessions) performing the goal-directed long-distance outdoor flight task. We used a wearable data recording device to synchronously capture the neural signals from the pigeon’s Hp and location information [33]. For the analysis, each trajectory was divided into three stages [32,34]:

1. Initial decision-making phase (DM): This phase reflects the pigeons’ decision-making process before navigation and includes recordings within a radius of 300 m from the release site.
2. En route navigation phase (ER): This phase represents the pigeons’ navigation process toward home and includes all recordings between the DM and LN stages (see below).
3. Local navigation around home (LN): This stage signifies the pigeons’ flight near the loft and includes recordings within a radius of 200 m from the loft.

During the flight process, neural signals and location data were collected synchronously by a wearable data recording device with a weight of 20 g. This device consisted of a neural signal acquisition module (ADS1299 with a sample rate of 1 kHz, 8 channels, sampling accuracy: 0.1 μV), a GPS module (ATGM336H-5N with a sample rate of 10 Hz, positioning accuracy: 2.5 m CEP) and a 3.7 V lithium battery. The neural signal acquisition module was fixed at the electrode interface on the head, and the battery and GPS module were loaded in the backpack on the back. They were connected by soft wires.

The location data include the longitude, latitude, and speed of the pigeon during flight. Neural data and location data were synchronized and recorded in a multitasking manner. Synchronization was achieved by utilizing the system time recorded at each sampling, ensuring the alignment of neuro-recording and GPS data. To exclude the influence of animals searching for directions during the DM, this study primarily analyzed data from the ER and LN. The data during the pigeons’ flight were analyzed within a window of 1000 ms, removing the undesirable channels caused by intermittent electrical connection, electrode separation, or motion noise. We used GPS data to obtain the pigeon’s flying speed, calculating the average speed over a 1 s window.

### 2.4. Analysis of Phase–Amplitude Coupling

**Figure 3:**
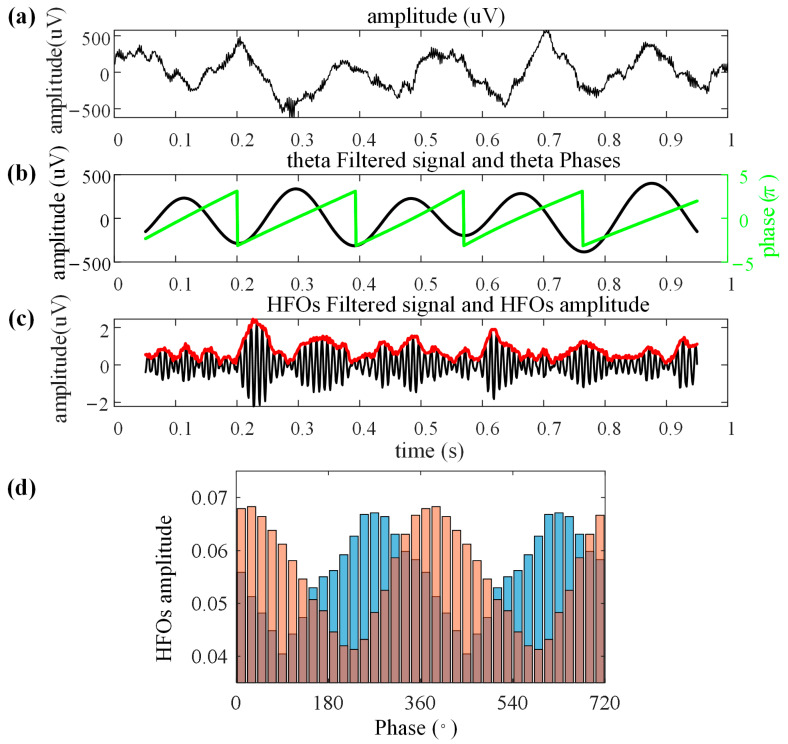
Steps in the computation of phase–amplitude distribution. (**a**) Raw local field potential signals were recorded from the hippocampus of pigeon. (**b**) Filtered theta band (3–6 Hz) signals and extracted low-frequency phases. (**c**) Filtered high-frequency oscillations (HFOs) band (120–160 Hz) signals (black line) and HFO amplitude (red line). (**d**) Two amplitude distributions (blue bar, orange bar, and tawny bar indicated the area where the two distributions overlap) with different phase bins.

In this study, we employed the modulation index (MI) as described by Tort et al. [11,35] to assess the strength of PAC within the Hp region. We also used correlation coefficients to evaluate the degree of the phase–amplitude “distribution” correlation. MI assesses whether the phase ($f_{p}$) of slower oscillations modulates the amplitude of faster oscillations ($f_{A}$). The calculation of MI proceeds as follows:

1. The raw signals are filtered to obtain theta-filtered signals and HFOs-filtered signals, from which the theta phase ($f_{p}$ and HFO amplitude ($f_{A}$) are extracted. To minimize the edge effects of the filtered signals, 50 ms of data is removed from both ends when extracting the phase and amplitude.
2. $f_{p}$ phase values are binned into intervals of 20 degrees, and the average amplitude ($f_{p}$) within each phase interval is calculated.
3. The mean amplitude ($f_{A}$) within each phase interval is normalized by dividing it by the sum of all mean amplitudes within the phase intervals, yielding the phase–amplitude “distribution” P. We have presented the above calculation procedure (Figure 3).
4. The MI is calculated using the Kullback–Leibler (KL) distance, which quantifies the deviation of P from a uniform distribution. Higher PAC results in greater deviation of the amplitude distribution from a uniform distribution. Finally, the KL distance values are normalized to a range from 0 to 1.
5. The above MI measures the distance between P and a uniform distribution but does not account for differences in the amplitude distribution phase across trials (Figure 3d) [36]. Hence, we employed the correlation coefficient to measure the differences between P across various trials. Specifically, we used the mean of all amplitude distributions for each pigeon during the same phase as the standard distribution for calculating the differences between trial distributions. For instance, during ground maze navigation, the mean of all amplitude distributions at S8 on path 1 for a pigeon served as the standard distribution to assess the correlation of amplitude distributions at different positions or on different navigation routes. During flight navigation, the mean of all amplitude distributions during the ER phase on path 1 was used as the standard distribution to gauge the correlation of amplitude distributions across different navigation stages.

These analyses are performed for all target frequencies. The phase frequencies of interest range from 2 to 20 Hz with a step size of 1 Hz, and the amplitude frequencies of interest range from 20 to 180 Hz with a step size of 5 Hz. To identify the frequency pairs of interest, we obtain coherence maps by plotting the MI for all frequency pairs on a two-dimensional pseudocolor plot. Here, the *x*-axis represents the phase modulation frequency, and the *y*-axis represents the amplitude frequency. For each epoch, the MI values were calculated for each frequency pair. The average MI value across all sessions for the same pigeon was taken to represent a single data sample. For each pigeon, we chose the channel with the highest average MI on the frequency pairs of interest for analysis.

### 2.5. Circular Histogram

The theta phase (6–8 Hz) corresponding to the occurrence of HFO peaks was extracted to create a circular histogram [37] using the phase–amplitude plot. Next, the CircHist toolbox was used to compute the average resultant vector (avgAng), the 95% confidence interval (95% CI), and the resultant vector length (r). The Rayleigh test from the CircStat toolbox was applied to assess the non-uniformity of circular data.

### 2.6. Statistical Test

All statistical analyses were performed using GraphPad Prism 9.0 software. The results are presented as mean ± standard deviation (std). Spearman correlation coefficients were used to measure correlations. To compare the differences in theta power, the PAC between different positions and paths, we used the non-parametric Kruskal–Wallis test with Dunn’s multiple pairwise comparisons for analysis. The non-parametric Mann–Whitney U-test was used to analyze differences between two stages in theta power, the MI, and corr. The statistically significant differences indicated by p values are as follows: * p<0.05, ** p<0.01, *** p<0.001, **** p<0.0001.

## 3. Results

### 3.1. Theta Phase Modulates HFOs in the Pigeon’s Hp during Maze Runs

**Figure 4:**
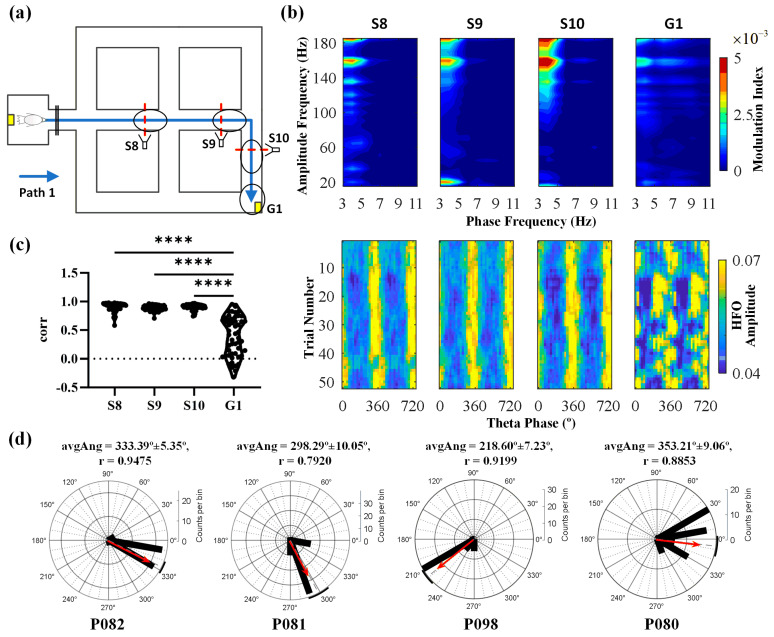
High-frequency oscillations (HFOs) amplitude modulation of local field potential (LFP) rhythms enabled by the theta phase in the hippocampus during maze runs. (**a**) An example trail for the experiment in the maze, with black ellipses indicating the spatial positions where neural signals were sampled and red markers denoting photobeam positions. (**b**) (Upper) phase–amplitude coupling calculated from LFP recorded in the Hp during maze navigation. The pseudocolor scale represents modulation index values. (Lower) pseudocolor scale representation of average HFO amplitudes as a function of theta phase for each trial. (**c**) Statistical analysis of similarity in HFO amplitudes within theta phase distributions (**** *p* < 0.0001). (**d**) Angular histograms of the hippocampal theta phases where the HFO peaks occurred. The black bars indicate the number of peaks of HFOs occurring in the corresponding theta phase. The red arrow indicates the direction (avgAng ± 95% CI) and magnitude (r) of the mean resultant vector. Rayleigh test was used to detect an unimodal deviation from uniformity (*p* < 0.01). The numbers under the diagrams indicate the four pigeons that carried out Task 1.

During navigation, we extracted neural signals for 1 s around four specific spatial positions (S8, S9, S10, G1, Figure 4a) to better visualize the relationship between oscillatory activity and different spatial locations. We found a theta-phase-to-HFO-amplitude modulation in the Hp in all pigeons. Taking pigeon P082 as an example, we observed that when the pigeon navigated through the maze, PAC occurred in its Hp, which disappeared near the goal location. Specifically, the theta phase (3–6 Hz) in the Hp primarily modulated the HFOs (140–180 Hz) (Figure 4b, upper). Based on comparing the average power spectral density (PSD) curves at different positions of the pigeon, it was seen that in the theta band, the PSD at the goal position was lower than at other positions that were in the active phase of the navigation process. We also analyzed the changes in theta power during navigation and showed that the power in the theta band was significantly higher during the active phase of the navigation process than at the G1 position (Supplementary Figure S2, Table S1). We calculated the phase–amplitude distributions between trials and used correlation measures to assess the distances between different trial distributions. The results indicated that the amplitude of HFOs in the Hp remained consistent with the theta phase during the navigation process across different trials (Figure 4b, lower). Additionally, there was a significant and consistent correlation in phase–amplitude distributions between the theta phase and HFO amplitude at positions S8, S9, and S10 during navigation and these distributions were significantly higher than those observed at the goal location (Kruskal–Wallis test; Kruskal–Wallis statistical value = 112.2, df = 3, *p* < 0.0001; Dunn’s test; S8 > G1; S9 > G1; S10 > G1; Figure 4c). Finally, we found stable phase preferences for individual hippocampal PAC but varying phase preferences across individuals (n = 4, trials = 188, Figure 4d).

Furthermore, we carried out a correlation analysis among the pigeon’s walking speed, theta power, HFO power, and MIs, respectively (Supplementary Material Table S2). There was no significant correlation between the strength of the PAC and the speed during the walking navigation (Spearman, P080: $r_{S}$ = −0.1306, *p* = 0.4916, n = 49; P081: $r_{S}$ = −0.0141, *p* = 0.9413, n = 45; P082: $r_{S}$ = 0.1202, *p* = 0.3959, n = 52; P098: $r_{S}$ = 0.0026, *p* = 0.9796, n = 42). Also, we found a significant correlation between the theta power and the speed in three out of four pigeons, while one did not exhibit such correlation (Spearman, P080: $r_{S}$ = 0.4297, *p* = 0.0178, n = 49; P081: $r_{S}$ = 0.5424, *p* = 0.0024, n = 45; P082: $r_{S}$ = −0.1403, *p* = 0.3211, n = 52; P098: $r_{S}$ = 0.2380, *p* = 0.0216, n = 42).

### 3.2. Hippocampal Theta–HFO Coupling Is Independent of Navigation Path and Goal

**Figure 5:**
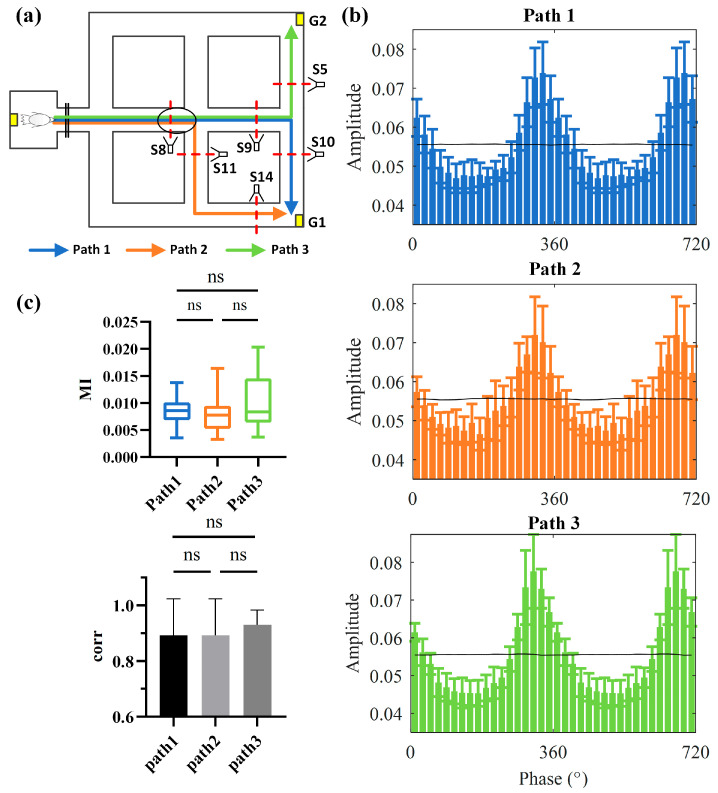
Hp theta–high-frequency oscillation (HFO) coupling in pigeons was independent of the navigation path or goal. (**a**) Three navigational paths were taken by pigeons to reach the goal positions. Paths 1 and 2 corresponded to the same navigation goal, while Path 3 had a different goal but shared some overlapping routes with Paths 1 and 2. The black ellipse showed the spatial position of the sampled neural signal. (**b**) Phase–amplitude distribution map of HFOs for P082, with the black line representing the average phase–amplitude distribution for random data. (**c**) Comparative analysis of theta–HFO coupling strength and similarity in phase–amplitude distributions under different navigation paths and goals.

We compared the PAC characteristics within the same decision location (S8) under different navigation paths and goals to test whether navigation path decisions and the goal location would affect the coupling between the Hp theta phase and HFOs (Figure 5a). We observed that the distribution of HFO amplitude under the Hp theta phase remained relatively stable across different navigation paths and different navigation goals (Figure 5b). We used the MI and correlations between distributions to assess the performance of PAC and differences between trials. The results indicated that there were no significant differences in the MI under different navigation paths and goals (Kruskal–Wallis test; Kruskal–Wallis statistical value = 5.066, df = 2, *p* = 0.0794; Figure 5c, upper). The distribution of HFO amplitude under theta phase showed high correlations between trials, and these correlations did not significantly differ across different paths and goals (Kruskal–Wallis test; Kruskal–Wallis statistical value = 1.073, df = 2, *p* = 0.5849; Figure 5c, lower). These findings suggest that theta phase–HFO coupling was not influenced by changes in navigation paths or goals during the goal-directed process.

### 3.3. Theta Phase Modulates the Beta2 Amplitude during Flight Navigation

**Figure 6:**
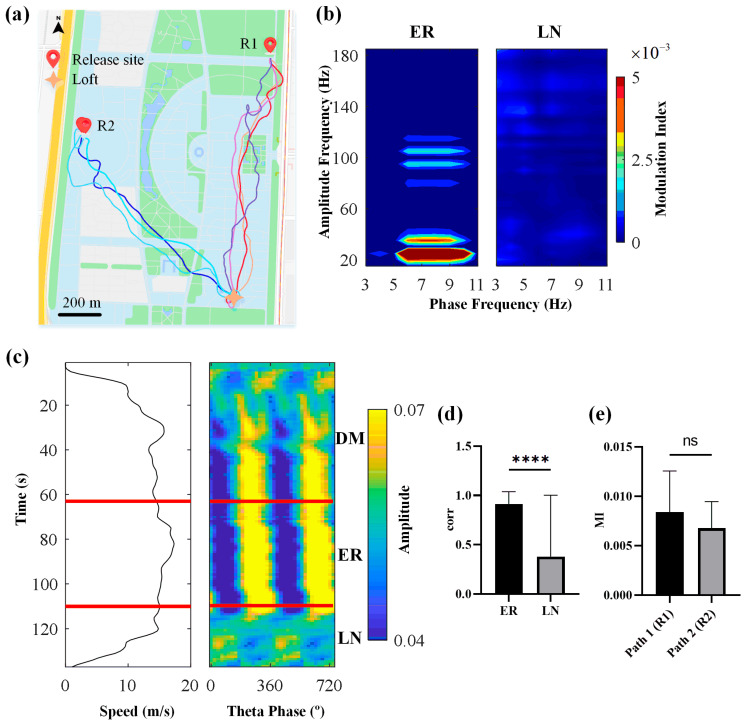
Phase-to-amplitude modulation in the Hp during outdoor flight. (**a**) A pigeon’s homing navigation trajectory at two release sites. (**b**) The mean phase-to-amplitude comodulograms were obtained from recorded local field potentials (LFPs) during different navigation phases. (**c**) Pseudocolor scale representation of beta2 amplitude as a function of theta phase during a complete homing flight of a pigeon, with the black line on the left representing the corresponding real-time speed and the red lines indicating boundaries between different phases. (**d**) Comparison of the correlation in beta2 amplitude distribution between en route navigation phase (ER) and local navigation around home (LN). (**e**) Comparison of modulation index (MI) along two navigational paths within the ER. Analyses were performed using the Mann–Whitney test with the significance level set at *p* < 0.05 (**** *p* < 0.0001; ns *p* > 0.05).

We showed a representative example of the typical flight trajectories of a pigeon at two release sites (Figure 6a). Two release sites in different directions drove the pigeon to form two different navigation paths. First, mean phase-to-amplitude comodulograms were created to visualize the coupling strength between the Hp theta phase and high-frequency oscillations. The results indicated that PAC occurred during the ER and disappeared in the proximity of the LN, consistent with findings from ground maze navigation tasks. During the ER, hippocampal theta oscillations (5–10 Hz) dynamically modulated beta2 band oscillations (20–30 Hz) (Figure 6b). The average PSD curves of the ER and LN phases also showed that the power of the LN phase was lower than the ER phase in the theta band (Supplementary Material Figure S3). The results showed that three of the four pigeons that performed flight navigation showed that theta power in the ER phase was significantly higher than in the LN phase (Mann–Whitney test, P010: *U* = 78317, *Z* = 8.91635, *p* < 0.0001, P014, *U* = 57457, *Z* = 4.99539, *p* = < 0.0001, P015: *U* = 13860, *Z* = −0.28887, *p* = 0.77297, P017: *U* = 75238, *Z* = 3.09866, *p* = 0.00192). Taking P014 as an example, we showed the pseudocolor scale representation of beta2 amplitude as a function of the theta phase during a complete homing flight, illustrating the dynamic changes in amplitude distribution during the flight process (Figure 6c). This result indicated that during the ER, the amplitude of the beta2 band in the Hp was concentrated at specific theta phases. An analysis of the beta2 amplitude distribution correlation revealed that it was significantly higher during the ER compared to the LN (Mann–Whitney test, *U* = 3578, *Z* = 5.63095, *p* < 0.0001, Figure 6d). Subsequently, we compared the influence of different flight paths from release sites R1 and R2 on the PAC strength during the ER. The results showed that there was no significant difference between Path 1 and Path 2 (Mann–Whitney test, *U* = 3003, *Z* = 1.73874, *p* = 0.0819, Figure 6e).

In addition, we analyzed the correlation of flight speed, MI, theta power, and HFO power in the ER phase of flight navigation (Supplementary Material Table S3). The results showed that there was no significant correlation between speed and MI in all pigeons (Spearman; P010: $r_{S}$ = 0.03531, *p* = 0.4642, n = 432; P014: $r_{S}$ = −0.08638, *p* = 0.0573, n = 485; P015: $r_{S}$ = 0.09031, *p* = 0.2701, n = 151; P017: $r_{S}$ = 0.05082, *p* = 0.3516, n = 338). Also, there was a slight tendency for the theta power to increase with speed during the ER (Spearman; P010: $r_{S}$ = 0.1950, *p* < 0.0001, n = 432; P014: $r_{S}$ = 0.2061, *p* < 0.0001, n = 485; P015: $r_{S}$ = −0.3141, *p* < 0.0001, n = 151; P017: $r_{S}$ = 0.6496, *p* < 0.0001, n = 338). However, the results showed that the PAC did not show a consistently significant correlation with the theta power and HFO power across all individuals.

## 4. Discussion

Our results were consistent with our initial prediction that the avian Hp exhibited PAC during navigation. During ground navigation, the hippocampal theta phase (3–6 Hz) primarily modulated the amplitude of high-frequency oscillations (140–180 Hz). While during flight navigation, the hippocampal theta phase (5–10 Hz) primarily modulated the amplitude of beta2 oscillations (20–40 Hz). Our correlation analyses between behavioral and neural data revealed that MI was independent of speed during walking and flying navigation, although theta power showed a significant dependence on speed. Furthermore, changes in navigation paths and goals did not affect PAC.

The finding that PAC in the avian Hp emerged during the active phase of navigation and tended to disappear during the goal-arrival phase was very consistent with the PAC of theta-phase modulation in the mammalian hippocampus [11,14]. Also, the significant correlation of hippocampal theta power with locomotor speed in birds was consistent with that in mammals [38,39]. The results provide support for the homology of avian and mammalian Hp and suggest that they may share similar mechanisms for navigational information processing. Associating the names to anatomical structures was not easy due to discrepancies between published boundaries of the avian hippocampus [40,41,42]. Using anatomical tracing and forebrain landmarks [43,44], our recording locations were all in the hippocampal formation (HF). Some recording locations were in the dorsolateral division (DL) of the HF or the hippocampus (Supplementary Material Figure S1). As far as it is known, cell type, connectivity, and functionality of the HF in birds largely match those of mammals [43,44,45,46]. In some hippocampal reports of food-caching birds, oscillations in the theta band did not seem to be observed [23]. This also raised the question of whether theta oscillations were not universal across all birds, but this could be due to differences in experimental techniques such as recording locations.

The peak phase of high-frequency amplitudes modulated by the theta phase was consistent across the same bird, while different phase preferences were expressed across individual birds. The consistency of the peak phase may be related to the task being performed. Some researchers suggested that this cross-frequency synchronization serves as an encoding scheme used by the Hp to organize the retrieval of impending memories. The theta rhythm provides an absolute phase reference necessary for encoding sequential information [47]. Another possibility is that the brain filters relevant information through the firing pattern of theta rhythm at a specific phase during the performance of a given task [48]. We speculated that the varying phase preferences observed between individuals may be attributed to several factors, including individual recording locations, different tuning properties of neurons involved in theta wave generation, and potential structural differences in the brain. Different hippocampal neurons were found to be locked to different theta phases in rodents and humans [49,50]. At the same time, there were significant phase differences when interregional CFC occured [51]. Subsequently, we found that after changing the navigation path and goal, this PAC phenomenon remained unchanged, implying that PAC appears to be specifically related to the progression of navigation. These findings indicate that dynamic, frequency-specific coupling is at least partially associated with the ongoing cognitive demands during the navigation process.

Under active goal-directed behavioral conditions, PAC emerges as a significant feature in LFP activity within the avian HF. These dynamic phase–amplitude modulations vary across different high-frequency bands and sub-frequency bands within the theta range during different behavioral patterns. During ground-based navigation, pigeons exhibit a dynamic modulation of 140–180 Hz HFOs via the 3–6 Hz theta sub-band. In contrast, during flight-based navigation, the 5–10 Hz theta sub-band dynamically modulates the 20–30 Hz beta2 band. The differences in coupling frequency bands may be attributed to different modes of locomotion, large speed differences, or different spatial scales. During navigation across different spatial scales, bat hippocampal place cells exhibit multiple place fields and representations across multiple scales [52,53]. This multiscale encoding forms the foundational representation for all environmental scales. Is the difference in high-frequency amplitude modulation of PAC during flight-based navigation attributed to the multiscale representations in the HF? One potential possibility is that the Hp needs to integrate different sensory cues in different spatial scales [24], resulting in differences between coupled bands.

In this study, we found that there was no significant correlation between the MI and the speed of movement. However, speed and theta power were significantly correlated. This phenomenon seems to support a form of “phase coded” information that is used to convey information from neural computation [11]. Although significant PAC has not been found in the bats’ Hp due to the lack of sequential theta oscillations [54], the amplitude of gamma-band activity couples with the phase of low-frequency LFPs in both the frontal and auditory cortex [55]. This form seemed to increase the possibility that the high-frequency occurrence of theta modulation was actively acquiring sequential information, while suggesting that PAC may be a shared information processing mechanism across different species.

The difficulty of obtaining valid data in pigeon flight navigation limits the analysis of hippocampal electrophysiology and behavior during flight. Due to the intense motion caused by pigeon flight and the limitation of the number of releases per day, as well as the inability to view the recorded signals in real time, we were unable to collect valid data every time. Differences in the positions of Hp and HF may also have an effect on the PAC. The preferred phases of phase–amplitude distributions in different individuals can also be explored more extensively when signals can be acquired from electrode arrays of a sufficiently large area. Some reports have also emphasized the effect of the degree of learning on PAC in mammals [12]. However, our study was conducted in the context of learned navigation, ignoring the influence of the learning process. In pigeon homing flights, we observed that pigeons did not fly towards the nest immediately after release from the release site, but hovered above the release site for several weeks as if determining/searching for direction. We also did not consider pigeons exploring the environment/determining direction in the ground navigation experiments. Thus, the activity in the hippocampal area at the start of navigation deserves further in-depth study. Also, the relevance of the neural patterns of the avian Hp to navigational behavior should be further investigated, since birds have two completely different locomotor modes: flying and walking.

## 5. Conclusions

In this study, we investigated the interactions between different frequency bands in the HF of pigeons during ground-based maze and outdoor flight navigation. We observed that the high-frequency amplitude in the HF of a pigeon was modulated by the theta phase during the navigation in two different modes of locomotion, with this modulation weakening as the pigeon approached the goal locations. At the same time, the MI and amplitude distribution showed no significant differences between different navigation paths and goals, suggesting that this coupling may be more associated with the navigational processes with ongoing cognitive demands. The correlation analysis between the MI and movement speed showed that the pigeon’s speed did not significantly impact the strength of PAC in both walking and flying movement modes, although theta power showed a significant dependence on speed. In conclusion, our results provide evidence of the hippocampal CFC of pigeons during navigation in different locomotion modes. Our findings indicate that the avian HF may need to coordinate activities across multiple frequency bands to integrate various cues or spatial cognition during navigation. These findings provide evidence that birds and mammalian hippocampal regions share similar information processing mechanisms during navigation and also contribute to understanding the potential mechanisms of multi-frequency information interaction in the HF.

## Data Availability

The datasets analyzed in the current study are available from the corresponding author on reasonable request.

## Supplementary Materials

The following supporting information can be downloaded at: https://www.mdpi.com/article/10.3390/ani14030439/s1, Figure S1: Illustration of implanting locations. (a) The electrode track reconstruction. triangle: Pigeons performing walking navigation; Round: Pigeons performing flying navigation; Hp: Hippocampus. (b) An example of the electrode tracks; Figure S2: The analysis of LFP at four different positions during Task 1. (a) Mean power spectrum curves of four posi-tions in Task 1 (delta: 1–3 Hz, theta: 4–12 Hz, beta: 13–30 Hz, gamma: 31–100 Hz, HFOs: 100–180 Hz, mean ± std). (b) Average theta (3–6 Hz) power of Hp as a percentage of the overall power of LFP at four different positions for four pigeons performing Task1. (c) The mean MI of four pigeons performing Task 1 (mean ± std). (d) The correlation of the amplitude distribution of pigeons in different positions (mean ± std); Table S1: Differences in theta power at four different locations. Kruskal–Wallis test and pairwise Dunn tests were used in statistical analyses. The significance level in pairwise Dunn comparison set at *p* < 0.05; Figure S3: The analysis of LFP at two phases during Task 2. (a) Mean power spectrum curves of two phases during Task 2 (delta: 1–3 Hz, theta: 4–12 Hz, beta: 13–30 Hz, gamma: 31–100 Hz, HFOs: 100–180 Hz, mean ± std). (b) Average theta (5–10 Hz) power of Hp as a percentage of the overall power of LFP at two phases for four pigeons performing Task 2. (c) The mean MI of four pigeons performing Task 2 (mean ± std); Figure S4: Average theta (3–10 Hz) power of Hp as a percentage of the overall power of LFP in both two tasks (pigeons in Task 1: P080, P081, P082, P098; pigeons in Task 2: P010, P014, P015, P017). The boxes extend from the 25th to 75th percentiles. The whiskers denote minimum and maximum values; Table S2: Correlation analysis of PAC influencing factors for each pigeon during task 1; Table S3: Correlation analysis of PAC influencing factors for each pigeon during task 2.

## References

[1] Chersi F., Burgess N. (2015). The Cognitive Architecture of Spatial Navigation: Hippocampal and Striatal Contributions. Neuron.

[2] Johnson A., Redish A.D. (2007). Neural ensembles in CA3 transiently encode paths forward of the animal at a decision point. J. Neurosci..

[3] Eichenbaum H. (2017). The role of the hippocampus in navigation is memory. J. Neurophysiol..

[4] Bingman V.P., Gagliardo A., Hough G.E., Ioale P., Kahn M.C., Siegel J.J. (2005). The avian hippocampus, homing in pigeons and the memory representation of large-scale space. Integr. Comp. Biol..

[5] Jeewajee A., Lever C., Burton S., O’Keefe J., Burgess N. (2008). Environmental novelty is signaled by reduction of the hippocampal theta frequency. Hippocampus.

[6] Kunz L., Wang L., Lachner-Piza D., Zhang H., Brandt A., Dumpelmann M., Reinacher P.C., Coenen V.A., Chen D., Wang W.X. (2019). Hippocampal theta phases organize the reactivation of large-scale electrophysiological representations during goal-directed navigation. Sci. Adv..

[7] Schroeder C.E., Lakatos P. (2009). Low-frequency neuronal oscillations as instruments of sensory selection. Trends Neurosci..

[8] Jackson J., Goutagny R., Williams S. (2011). Fast and slow gamma rhythms are intrinsically and independently generated in the subiculum. J. Neurosci..

[9] Cole S.R., Voytek B. (2017). Brain Oscillations and the Importance of Waveform Shape. Trends Cogn. Sci..

[10] Nguyen Chi V., Muller C., Wolfenstetter T., Yanovsky Y., Draguhn A., Tort A.B., Brankack J. (2016). Hippocampal Respiration-Driven Rhythm Distinct from Theta Oscillations in Awake Mice. J. Neurosci..

[11] Tort A.B., Kramer M.A., Thorn C., Gibson D.J., Kubota Y., Graybiel A.M., Kopell N.J. (2008). Dynamic cross-frequency couplings of local field potential oscillations in rat striatum and hippocampus during performance of a T-maze task. Proc. Natl. Acad. Sci. USA.

[12] Tort A.B., Komorowski R.W., Manns J.R., Kopell N.J., Eichenbaum H. (2009). Theta-gamma coupling increases during the learning of item-context associations. Proc. Natl. Acad. Sci. USA.

[13] Wang W. (2021). Brain network features based on theta-gamma cross-frequency coupling connections in EEG for emotion recognition. Neurosci. Lett..

[14] Tavares L.C.S., Tort A.B.L. (2022). Hippocampal-prefrontal interactions during spatial decision-making. Hippocampus.

[15] Belluscio M.A., Mizuseki K., Schmidt R., Kempter R., Buzsaki G. (2012). Cross-frequency phase-phase coupling between theta and gamma oscillations in the hippocampus. J. Neurosci..

[16] Salimpour Y., Anderson W.S. (2019). Cross-Frequency Coupling Based Neuromodulation for Treating Neurological Disorders. Front. Neurosci..

[17] Vivekananda U., Bush D., Bisby J.A., Baxendale S., Rodionov R., Diehl B., Chowdhury F.A., McEvoy A.W., Miserocchi A., Walker M.C. (2021). Theta power and theta-gamma coupling support long-term spatial memory retrieval. Hippocampus.

[18] Scheffer-Teixeira R., Belchior H., Caixeta F.V., Souza B.C., Ribeiro S., Tort A.B. (2012). Theta phase modulates multiple layer-specific oscillations in the CA1 region. Cereb. Cortex.

[19] Jacobs J., Zelmann R., Jirsch J., Chander R., Dubeau C.E., Gotman J. (2009). High frequency oscillations (80-500 Hz) in the preictal period in patients with focal seizures. Epilepsia.

[20] Colgin L.L. (2016). Rhythms of the hippocampal network. Nat. Rev. Neurosci..

[21] Buzsaki G. (2015). Hippocampal sharp wave-ripple: A cognitive biomarker for episodic memory and planning. Hippocampus.

[22] Takeuchi S., Mima T., Murai R., Shimazu H., Isomura Y., Tsujimoto T. (2015). Gamma Oscillations and Their Cross-frequency Coupling in the Primate Hippocampus during Sleep. Sleep.

[23] Payne H.L., Lynch G.F., Aronov D. (2021). Neural representations of space in the hippocampus of a food-caching bird. Science.

[24] Mouritsen H., Heyers D., Gunturkun O. (2016). The Neural Basis of Long-Distance Navigation in Birds. Annu. Rev. Physiol..

[25] Siegel J.J., Nitz D., Bingman V.P. (2000). Hippocampal theta rhythm in awake, freely moving homing pigeons. Hippocampus.

[26] Dotson N.M., Yartsev M.M. (2021). Nonlocal spatiotemporal representation in the hippocampus of freely flying bats. Science.

[27] Nunez A., Buno W. (2021). The Theta Rhythm of the Hippocampus: From Neuronal and Circuit Mechanisms to Behavior. Front. Cell. Neurosci..

[28] Vyssotski A.L., Dell’Omo G., Dell’Ariccia G., Abramchuk A.N., Serkov A.N., Latanov A.V., Loizzo A., Wolfer D.P., Lipp H.P. (2009). EEG responses to visual landmarks in flying pigeons. Curr. Biol..

[29] Li M., Cheng S., Fan J., Shang Z., Wan H., Yang L., Yang L. (2022). Disarrangement and reorganization of the hippocampal functional connectivity during the spatial path adjustment of pigeons. BMC Zool..

[30] Harvey J., Karten W.H. (1967). A Stereotaxic Atlas of the Brain of the Pigeon (Columba livia).

[31] Li M., Fan J., Lin L., Shang Z., Wan H. (2022). Elevated Gamma Connectivity in Nidopallium Caudolaterale of Pigeons during Spatial Path Adjustment. Animals.

[32] Gagliardo A., Pollonara E., Wikelski M. (2020). Pigeons remember visual landmarks after one release and rely upon them more if they are anosmic. Anim. Behav..

[33] Wang L., Yang L., Li M., Yang Z., Shang Z. Development of wearable miniature neural signal recording system for birds. Proceedings of the 2020 Chinese Automation Congress (CAC).

[34] Gagliardo A., Colombo S., Pollonara E., Casini G., Rossino M.G., Wikelski M., Bingman V.P. (2021). GPS-profiling of retrograde navigational impairments associated with hippocampal lesion in homing pigeons. Behav. Brain Res..

[35] Tort A.B., Komorowski R., Eichenbaum H., Kopell N. (2010). Measuring phase-amplitude coupling between neuronal oscillations of different frequencies. J. Neurophysiol..

[36] Li Z., Bai X., Hu R., Li X. (2021). Measuring Phase-Amplitude Coupling Based on the Jensen-Shannon Divergence and Correlation Matrix. IEEE Trans. Neural Syst. Rehabil. Eng..

[37] Zittrell F. CircHist: Circular Histogram in MATLAB. https://zenodo.org/records/3971901.

[38] Kennedy J.P., Zhou Y., Qin Y., Lovett S.D., Sheremet A., Burke S.N., Maurer A.P. (2022). A Direct Comparison of Theta Power and Frequency to Speed and Acceleration. J. Neurosci..

[39] Zahra M.A., Schuette P., Fields T.A., Tran M.E., Siddiqui S.M., Hasulak N.R., Tcheng T.K., Eliashiv D., Mankin E.A., Stern J. (2017). Theta Oscillations in the Human Medial Temporal Lobe during Real-World Ambulatory Movement. Curr. Biol..

[40] Applegate M.C., Gutnichenko K.S., Mackevicius E.L., Aronov D. (2023). An entorhinal-like region in food-caching birds. Curr. Biol..

[41] Reiner A., Perkel D.J., Bruce L.L., Butler A.B., Csillag A., Kuenzel W., Medina L., Paxinos G., Shimizu T., Striedter G. (2004). Revised nomenclature for avian telencephalon and some related brainstem nuclei. J. Comp. Neurol..

[42] Atoji Y., Wild J.M. (2005). Afferent and efferent connections of the dorsolateral corticoid area and a comparison with connections of the temporo-parieto-occipital area in the pigeon (*Columba livia*). J. Comp. Neurol..

[43] Herold C., Coppola V.J., Bingman V.P. (2015). The maturation of research into the avian hippocampal formation: Recent discoveries from one of the nature’s foremost navigators. Hippocampus.

[44] Bingman V.P., Muzio R.N. (2017). Reflections on the Structural-Functional Evolution of the Hippocampus: What Is the Big Deal about a Dentate Gyrus?. Brain Behav. Evol..

[45] Herold C., Schlomer P., Mafoppa-Fomat I., Mehlhorn J., Amunts K., Axer M. (2019). The hippocampus of birds in a view of evolutionary connectomics. Cortex.

[46] Herold C., Ockermann P.N., Amunts K. (2022). Behavioral Training Related Neurotransmitter Receptor Expression Dynamics in the Nidopallium Caudolaterale and the Hippocampal Formation of Pigeons. Front. Physiol..

[47] Lisman J. (2005). The theta/gamma discrete phase code occuring during the hippocampal phase precession may be a more general brain coding scheme. Hippocampus.

[48] Jensen O. (2005). Reading the hippocampal code by theta phase-locking. Trends Cogn. Sci..

[49] O’Keefe J., Recce M.L. (1993). Phase relationship between hippocampal place units and the EEG theta rhythm. Hippocampus.

[50] Jacobs J., Kahana M.J., Ekstrom A.D., Fried I. (2007). Brain oscillations control timing of single-neuron activity in humans. J. Neurosci..

[51] Atiwiwat D., Aquilino M., Devinsky O., Bardakjian B.L., Carlen P.L. (2023). Interregional phase-amplitude coupling between theta rhythm in the nucleus tractus solitarius and high-frequency oscillations in the hippocampus during REM sleep in rats. Sleep.

[52] Eliav T., Maimon S.R., Aljadeff J., Tsodyks M., Ginosar G., Las L., Ulanovsky N. (2021). Multiscale representation of very large environments in the hippocampus of flying bats. Science.

[53] Kjelstrup K.B., Solstad T., Brun V.H., Hafting T., Leutgeb S., Witter M.P., Moser E.I., Moser M.B. (2008). Finite scale of spatial representation in the hippocampus. Science.

[54] Ulanovsky N., Moss C.F. (2007). Hippocampal cellular and network activity in freely moving echolocating bats. Nat. Neurosci..

[55] Garcia-Rosales F., Lopez-Jury L., Gonzalez-Palomares E., Cabral-Calderin Y., Kossl M., Hechavarria J.C. (2022). Phase-amplitude coupling profiles differ in frontal and auditory cortices of bats. Eur. J. Neurosci..

