# Supplementary material for: Phase–Amplitude Coupling between Theta Rhythm and High-Frequency Oscillations in the Hippocampus of Pigeons during Navigation
Source: Animals (Basel). 2024 Jan 29;14(3):439. doi: 10.3390/ani14030439 (PMC10854523; doi:10.3390/ani14030439)
Supplement: Supplementary file 1 [file animals-14-00439-s001.zip › animals-2750087-supplementary.pdf]

Histology

After completion of all electrophysiological signal acquisition experiments, the electrode locations of all pigeons were marked with electrical stimulation (2 mA for 30 s). Each pigeon was euthanized by being injected with a lethal dose of pentobarbital via the chest muscle. Pigeons were perfused intracardially with saline and 4% paraformaldehyde, after which the brains were removed and fixed in 4% paraformaldehyde for 48 hours. Frozen coronal sections (50 μm) were collected from the electrode locations and then mounted on slides for dehydration, cleaning, staining, and imaging. Recording electrode tracks were reconstructed using the tissue damage observed histologically to verify the recording locations.

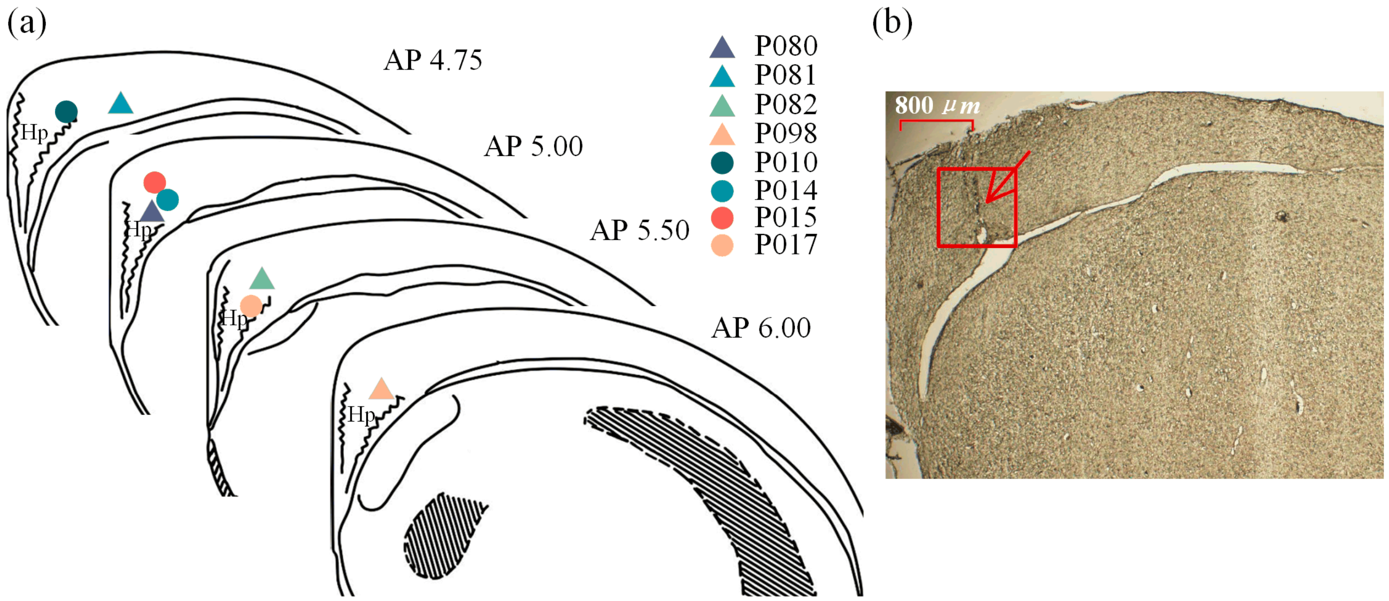

Figure S1. Illustration of implanting locations. (a) The electrode track reconstruction. triangle: Pigeons performing walking navigation; Round: Pigeons performing flying navigation; Hp: Hippocampus. (b) An example of the electrode tracks.

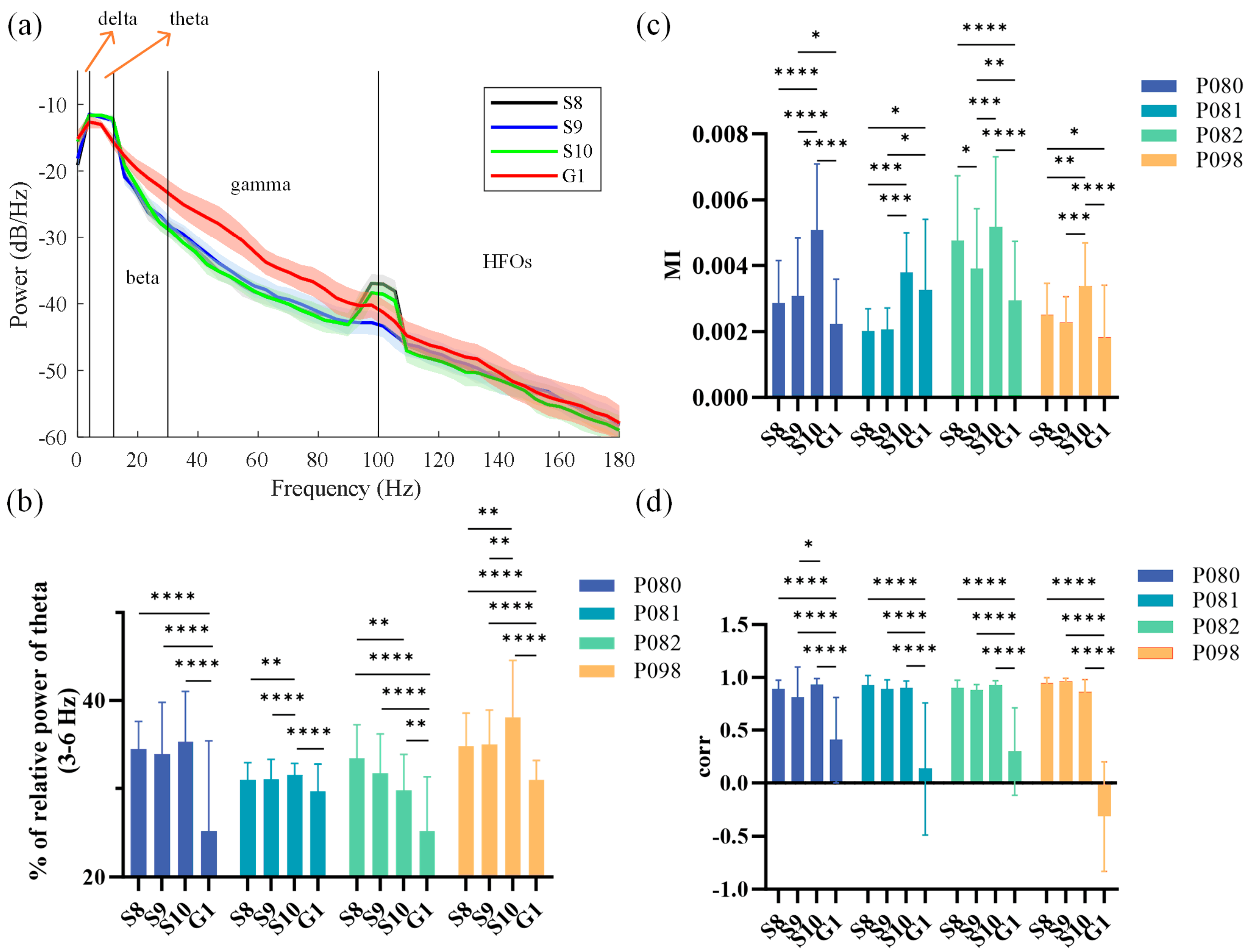

Figure S2. The analysis of LFP at four different positions during Task 1. (a) Mean power spectrum curves of four positions in Task 1(delta: 1-3 Hz, theta: 4-12 Hz, beta: 13-30 Hz, gamma: 31-100 Hz, HFOs: 100-180 Hz, mean ± std). (b) Average theta (3-6 Hz) power of Hp as a percentage of the overall power of LFP at four different positions for four pigeons performing Task1. (c) The mean MI of four pigeons performing Task 1 (mean ± std). (d) The correlation of the amplitude distribution of pigeons in different positions (mean ± std).

Table S1. Differences in theta power at four different locations. Kruskal–Wallis test and pairwise Dunn tests were used in statistical analyses. The significance level in pairwise Dunn comparison set at $p < 0.05$.

	P080	P081	P082	P098
Position				
S8	0.344±0.032	0.310±0.020	0.334±0.039	0.347±0.038
S9	0.339±0.059	0.310±0.023	0.317±0.044	0.349±0.040
S10	0.353±0.058	0.315±0.013	0.298±0.041	0.381±0.064
G1	0.251±0.102	0.297±0.031	0.252±0.061	0.309±0.022
n	49	45	52	42
Df	3	3	3	3
Kruskal-Wallis statisical value	53.98	39.90	47.64	64.40
p-value	<0.0001	<0.0001	<0.0001	<0.0001

Pairwise adjusted significant differences, Dunn test	S8 > G1<br>S9 > G1<br>S10 > G1	S10 > G1	S8 > G1<br>S9 > G1<br>S10 > G1	S8 > G1<br>S9 > G1<br>S10 > G1

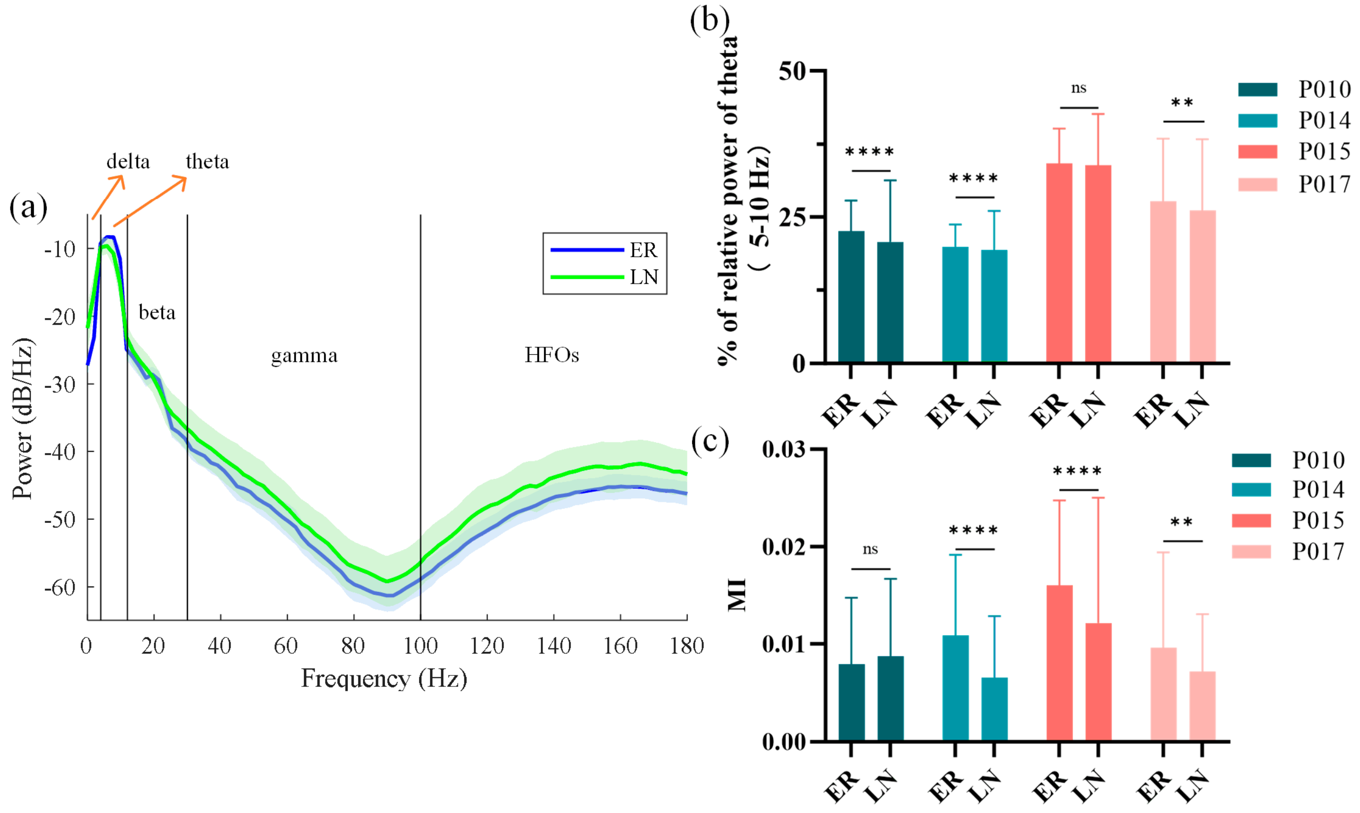

Figure S3. The analysis of LFP at two phases during Task 2. (a) Mean power spectrum curves of two phases during Task 2 (delta: 1-3 Hz, theta: 4-12 Hz, beta: 13-30 Hz, gamma: 31-100 Hz, HFOs: 100-180 Hz, mean ± std). (b) Average theta (5-10 Hz) power of Hp as a percentage of the overall power of LFP at two phases for four pigeons performing Task 2. (c) The mean MI of four pigeons performing Task 2 (mean ± std).

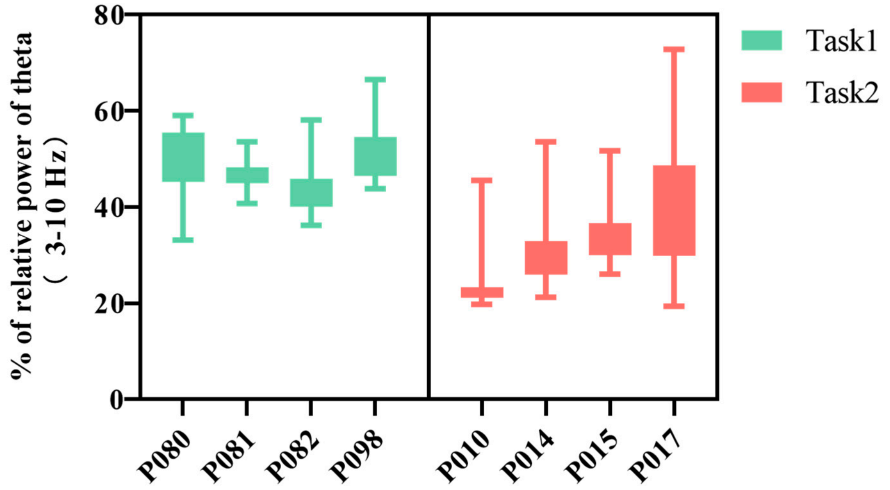

Figure S4. Average theta (3-10 Hz) power of Hp as a percentage of the overall power of LFP in both two tasks (pigeons in Task 1: P080, P081, P082, P098; pigeons in Task 2: P010, P014, P015, P017). The boxes extend from the 25th to 75th percentiles. The whiskers denote minimum and maximum values.

Table S2. Correlation analysis of PAC influencing factors for each pigeon during task 1.

Pigeon	Speed vs MI	Theta vs MI	HFO vs MI	Speed vs theta	Speed vs HFO
P080	**ns**	ns	ns	*	ns
P081	**ns**	ns	ns	**	***
P082	**ns**	**	*	**ns**	ns
P098	**ns**	****	****	*	ns

Table S3. Correlation analysis of PAC influencing factors for each pigeon during task 2.

Pigeon	Speed vs MI	Theta vs MI	Beta2 vs MI	Speed vs theta	Speed vs Beta2
P010	**ns**	*	ns	****	****
P014	**ns**	****	****	****	**
P015	**ns**	ns	*	****	**
P017	**ns**	ns	ns	****	ns
